# Justified Concern or Exaggerated Fear: The Risk of Anaphylaxis in Percutaneous Treatment of Cystic Echinococcosis—A Systematic Literature Review

**DOI:** 10.1371/journal.pntd.0001154

**Published:** 2011-06-14

**Authors:** Andreas Neumayr, Giuliana Troia, Chiara de Bernardis, Francesca Tamarozzi, Sam Goblirsch, Luca Piccoli, Christoph Hatz, Carlo Filice, Enrico Brunetti

**Affiliations:** 1 Swiss Tropical and Public Health Institute, Basel, Switzerland; 2 Division of Infectious and Tropical Diseases, University of Pavia, IRCCS S. Matteo Hospital Foundation, WHO Collaborating Centre for Clinical Management of Cystic Echinococcosis, Pavia, Italy; 3 Department of Medicine and Pediatrics, University of Minnesota, Minneapolis, Minnesota, United States of America; 4 Ultrasound Unit, Department of Infectious Diseases, University of Pavia, IRCCS S. Matteo Hospital Foundation, WHO Collaborating Centre for Clinical Management of Cystic Echinococcosis, Pavia, Italy; London School of Hygiene & Tropical Medicine, United Kingdom

## Abstract

Percutaneous treatment (PT) emerged in the mid-1980s as an alternative to surgery for selected cases of abdominal cystic echinococcosis (CE). Despite its efficacy and widespread use, the puncture of echinococcal cysts is still far from being universally accepted. One of the main reasons for this reluctance is the perceived risk of anaphylaxis linked to PTs. To quantify the risk of anaphylactic reactions and lethal anaphylaxis with PT, we systematically searched MEDLINE for publications on PT of CE and reviewed the PT-related complications. After including 124 publications published between 1980 and 2010, we collected a total number of 5943 PT procedures on 5517 hepatic and non-hepatic echinococcal cysts. Overall, two cases of lethal anaphylaxis and 99 reversible anaphylactic reactions were reported. Lethal anaphylaxis occurred in 0.03% of PT procedures, corresponding to 0.04% of treated cysts, while reversible allergic reactions complicated 1.7% of PTs, corresponding to 1.8% of treated echinococcal cysts. Analysis of the literature shows that lethal anaphylaxis related to percutaneous treatment of CE is an extremely rare event and is observed no more frequently than drug-related anaphylactic side effects.

## Author Summary

The risk of anaphylactic shock is the objection most often raised by opponents of percutaneous treatments for cystic echinococcosis, but there are no updated figures on the actual occurrence of anaphylaxis as a complication of this treatment.

To assess the number of lethal and non-lethal anaphylactic reactions following percutaneous aspiration of echinococcal cysts, we systematically reviewed the literature published from 1980–2010.

The analysis of the available literature shows that the risk of severe anaphylactic reactions resulting from percutaneous treatment of echinococcal cysts has been widely exaggerated and the actual risk may be lower than that following administration of certain antibiotics.

Provided adequate stand-by resuscitation measures are available, each time an echinococcal cyst is punctured, fear of anaphylactic shock is no longer justified as an argument to avoid this therapeutic option.

## Introduction

Human cystic echinococcosis (CE), caused by the larval stage of the cestode *Echinococcus granulosus*, is a cosmopolitan parasitic zoonosis, affecting mainly the liver (∼70%) and the lung (∼20%) of the human intermediate host. Clinical symptoms depend on the location, number, and size of the cysts. Until anthelminthic chemotherapy became available (mebendazole in the 1970s and albendazole in the early 1980s), surgery was the only treatment choice. The spectrum of therapeutic options was further extended in the mid-1980s when the increasing availability of modern imaging techniques, namely ultrasound, allowed the introduction of image-guided percutaneous treatment (PT) methods.

Over the years, various PTs have been developed, based on the classic PAIR (Puncture of the cyst, Aspiration of the cyst fluid, Injection of a scolicidal agent, and Re-aspiration of the cyst content) procedure [1], [2] with minor variations of the essential steps [3]–[5]. Different catheterization techniques allowing aspiration of the solid content of cysts have also been developed for those cyst stages that are often unresponsive to PAIR [6], [7].

Despite the wide use of PTs in the last two and a half decades, the fear of anaphylactic shock and dissemination due to the spillage of cystic fluid is still quoted by physicians favoring surgery for the treatment of CE [8], [9]. However, anaphylactic reactions in CE occur not only as a side effect of PT, but also of surgical treatment [10]–[17], result of accidental trauma [18]–[20] and even spontaneously [21]–[24].

To our knowledge there are no updated figures on the frequency of anaphylactic reactions, anaphylactic shock or lethal anaphylaxis following PTs of echinococcal cysts. To quantify the risk of allergic reactions and lethal anaphylaxis related to PT of echinococcal cysts we systematically reviewed the published literature.

## Methods

**Figure 1:**
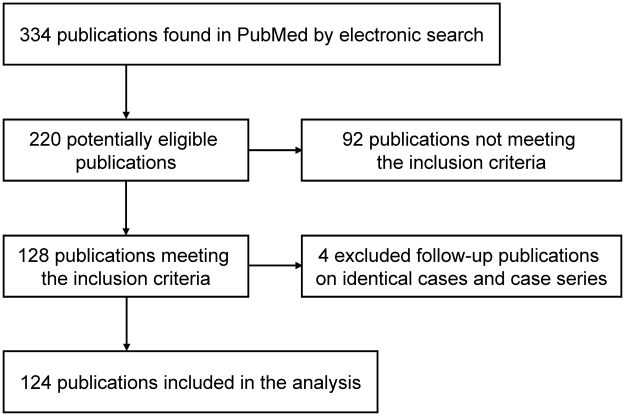
Flow chart of search and selection of eligible publications.

We performed a PubMed (MEDLINE) search of the literature using the key words “echinococcal cysts”, “hydatid cysts”, “cystic echinococcosis”, “hydatidosis”, “PAIR”, and “percutaneous treatment” and reviewed the available references published between January 1980 and December 2009 for eligible publications (Figure 1).

The inclusion criteria were as follows:

All publications on PT of *E. granulosus* cysts with information about the number of treated cysts, the number of PT procedures and the occurrence of lethal complications were included. When the original article was not obtainable but the abstract containing the requested information was, the publication was included in the analysis.

In some cases repeated PTs of the same echinococcal cysts were performed during the course of treatment. In these instances, we collected the total number of treated echinococcal cysts and the corresponding total number of PT procedures.

To avoid multiple counting (duplication) of identical procedures and cases, follow-up publications on identical procedures and cases were traced and excluded (references S1).

In human CE, the liver is the organ most frequently affected. Therefore, we divided the collected cases and PT procedures according to the anatomical location into “hepatic cysts” (Table S1) and “extra-hepatic cysts” (Table S2).

When the exact anatomical locations of the cysts were not specified, the data was collected separately (Table 1).

**Table 1:** Percutaneous treatment of hydatid cysts of various locations.

Authors	Year of publication	Journal	No. of percutaneous treated cysts in various locations	No. of percutaneous treated liver cysts	No. of puncture procedures	No. of reversible complications	No. of lethal complications
Gargouri et al.	1990	Cardiovasc Intervent Radiol.	120 *		120	7	
Wang et al.	1994	Zhongguo Ji Sheng Chong Xue Yu Ji Sheng Chong Bing Za Zhi	361 †		361	1	
Saenz-Santamaria	1995	Diagn Cytopathol	17 ‡		17		
Von Sinner et al.	1995	Acta Radiol	31 §		41	6	
Vuitton et al.	2002	GUT	351 #	1263	1789	409	
Total			880	1263	2328	423	0

*: “liver, peritoneum, spleen, kidney, muscle and bone”.

**†:** hepatic and abdominal hydatid cysts.

**‡:** no location specified.

§“abdomen, thorax, spine and bone”.

# abdominal hydatid cysts and “other locations”.

Information about the reported complications was collected accordingly, differentiated into “lethal complications” and “reversible complications” and summarized (Tables 2, 3, 4, 5, 6, 7).

**Table 2:** Overall lethal complications due to percutaneous treatment of hydatid cysts.

Lethal complications	No. of cases	% of treated hydatid cysts (n = 5517)	% of percutaneous treatment procedures (n = 5943)
Lethal anaphylactic shock *	2	0.04	0.03
Lethality related to percutaneous treatment procedure †	1	0.02	0.02
Lethality not related to percutaneous treatment procedure ‡	2	0.04	0.03
Total	5	0.09	0.08

*(Men et al.,1999; Giorgio et al., 2009).

**†:** (Vishnevskii et al., 1992).

**‡:** (Khuroo et al., 1991; Gavrilin et al., 2002).

**Table 3:** Reversible complications due to percutaneous treatment of hydatid liver cysts.

Reversible complications	No. of cases	% of treated liver hydatid cysts (n = 3232)	% of percutaneous treatment procedures (n = 3440)
Reversible severe anaphylactic reaction	4	0.12	0.12
Reversible mild anaphylactic reaction	2	0.06	0.06
Reversible anaphylactic reaction not specified	10	0.31	0.29
Allergic skin reaction (urticaria, rash, pruritus)	65	2.01	1.89
Fever	98	3.03	2.85
Hypotensive reaction	14	0.43	0.41
Vaso-vagal reaction	1	0.03	0.03
Nausea & vomiting	1	0.03	0.03
Cavity infection/abscess	59	1.83	1.72
Biliary fistula/rupture	35	1.08	1.02
Persisting drainage *	2	0.06	0.06
Peritoneal leakage	2	0.06	0.06
Subcapsular haematoma	6	0.19	0.17
Intracystic bleeding	1	0.03	0.03
Gallbladder haemorrhage	1	0.03	0.03
Active arterial haemorrhage †	1	0.03	0.03
Right-sided pleural effusion	13	0.40	0.38
Right-sided pneumothorax	1	0.03	0.03
Transient hypernatraemia ‡	2	0.06	0.06
Non-classified reversible complication	27	0.84	0.78
Total	345	10.66	10.05

*“Persisting drainage of serous fluid … was treated by keeping the drainage catheter in place until cessation of drainage (range 2–30 d) (Men et al., 1999).

**†:** Parenchymal liver laceration with active arterial haemorrhage from a branch of the right hepatic artery and the need for endovascular and surgical intervention (Loutfi et al., 2008).

**‡:** Due to the usage of hypertonic saline as scolecidal agent (Haddad et al., 2000).

**Table 4:** Reversible complications due to percutaneous treatment of hydatid cysts of various locations.

Reversible complications	No. of cases	% of treated hydatid cysts (n = 2143)	% of percutaneous treatment procedures (n = 2328)
Reversible moderate anaphylactic reaction	7	0.33	0.30
Reversible minor allergic reactions	5	0.23	0.21
Reversible anaphylactic reactions †	4	0.19	0.17
Reversible anaphylactic reactions ‡	1	0.05	0.04
Hypotensive reaction §	1	0.05	0.04
Fever	13	0.61	0.56
Bile leakage	392	18.29	16.84
Total	423	19.75	18.16

*published data is not specifying complications according to cyst location.

**†:** “facial oedema & acute dyspnoea” (Vuitton et al., 2002).

**‡:** unclassified reversible anaphylactic reaction (Wang et al., 1994).

**§:** (Von Sinner et al. 1995).

**Table 5:** Reversible complications due to percutaneous treatment of extra-hepatic hydatid cysts.

Reversible complications	No. of cases	% of treated non-hepatic hydatid cysts (n = 142)	% of percutaneous treatment procedures of non-hepatic cysts (n = 175)
Allergic skin reaction (urticaria)	1	0.70	0.57
“Fever & urticaria”	x *	-	-
Pneumothorax	1 †	0.70	0.57
Cavity infection/abscess	1	0.70	0.57
Non-classified reversible complication	6	4.23	3.43
Total	9	6.33	5.14

*“no complications were observed except…fever and urticaria”, no figures given (Zerem et al., 2005 [10 cases of percutaneous treated spleen hydatid cysts]).

**†:** Percutaneous treatment of a lung hydatid cyst (Gagal et al., 2005).

**Table 6:** Overall reversible complications due to percutaneous treatment of hydatid cysts.

Reversible complications	No. of cases	% of treated hydatid cysts (n = 5517)	% of percutaneous treatment procedures (n = 5943)
Reversible severe anaphylactic reaction	4	0.07	0.07
Reversible moderate anaphylactic reaction	7	0.13	0.12
Reversible mild/minor anaphylactic reaction	7	0.13	0.12
Reversible anaphylactic reaction not specified	15	0.27	0.25
Allergic skin reaction (urticaria, rash, pruritus)	66	1.20	1.11
“Fever & urticaria”	x *	-	-
Hypotensive reaction	15	0.27	0.25
Fever	111	2.01	1.87
Vaso-vagal reaction	1	0.02	0.02
Nausea & vomiting	1	0.02	0.02
Cavity infection/abscess	60	1.09	1.01
Biliary fistula/leakage/rupture	427	7.74	7.18
Persisting drainage †	2	0.04	0.03
Peritoneal leakage	2	0.04	0.03
Subcapsular haematoma	6	0.11	0.10
Intracystic bleeding	1	0.02	0.02
Gallbladder haemorrhage	1	0.02	0.02
Active arterial haemorrhage ‡	1	0.02	0.02
Right-sided pleural effusion	13	0.24	0.22
Pneumothorax	2	0.04	0.03
Transient hypernatraemia §	2	0.04	0.03
Non-classified reversible complication	33	0.60	0.56
Total	777	14.12	13.08

*“no complications were observed except…fever and urticaria”, no figures given (Zerem et al., 2005 [10 cases of percutaneous treated spleen hydatid cysts]).

**†:** “Persisting drainage of serous fluid … was treated by keeping the drainage catheter in place until cessation of drainage (range 2–30 d)” (Men et al., 1999).

**‡:** Parenchymal liver laceration with active arterial haemorrhage from a branch of the right hepatic artery and the need for endovascular and surgical intervention (Loutfi et al., 2008).

**§:** Due to the usage of hypertonic saline as scolecidal agent (Haddad et al., 2000).

**Table 7:** Overall reversible anaphylactic reactions due to percutaneous treatment of hydatid cysts.

Reversible anaphylactic complications	No. of cases	% of treated hydatid cysts (n = 5517)	% of percutaneous treatment procedures (n = 5943)
Reversible severe anaphylactic reaction	4	0.07	0.07
Reversible moderate anaphylactic reaction	7	0.13	0.12
Reversible mild/minor anaphylactic reaction	7	0.13	0.12
Reversible anaphylactic reaction not specified	15	0.27	0.25
Allergic skin reaction (urticaria, rash, pruritus)	66	1.20	1.11
“Fever & urticaria”	x *	-	-
Total	99	1.80	1.67

*“no complications were observed except…fever and urticaria”, no figures given (Zerem et al., 2005 [10 cases of percutaneous treated spleen hydatid cysts]).

It was impossible to retrospectively grade the severity of the reversible anaphylactic reactions due to the lack of a standardized definition of the events.

If the authors labelled subjective severity levels of the observed anaphylactic reactions (e.g. “severe”, “moderate”, “mild”, “minor”) we collected, summarized and listed them accordingly. In addition to the evaluation and quantification of anaphylactic reactions, we also collected and summarized other PT related complications, to allow a representative overview of all PT relevant complications.

## Results

One hundred-twenty-four publications met our inclusion criteria, with a total number of 5943 PT procedures performed for the diagnosis or treatment of 5517 echinococcal cysts. Ninety-two publications that did not meet the inclusion criteria were excluded from the analysis. Four publications were follow-up publications on identical cases or case series and therefore excluded from analysis.

In all but three of the publications included, detailed information about the observed reversible complications were available. In one additional publication, the observed complications were specified but not quantified. These four publications were labeled in the tables accordingly (Tables S1, S3, 5, 6, 7).

For 863 cysts, information concerning the organ location involved was available, but exact number of cysts for each organ was not. For 17 cysts, information about the location was not available. The publications covering these 880 cysts were labeled in the tables accordingly (Table 1).

A detailed analysis of the observed complications in reference to size, stage, and exact location within the affected organs was impossible due to lack of details in the original publications. Overall, five lethal and 777 reversible complications were collected (Tables 2, 6). Of the five lethal complications, three were related to the PT procedure, while two fatalities occurred due to PT “unrelated causes”. Of the three PT related fatalities, two lethal anaphylactic shocks and one fatality “associated with the use of the method” were reported. Unfortunately, detailed information about the two fatalities due to “unrelated causes” [25], [26] and the fatality reported as “associated with the use of the method” [27] were not obtainable.

There were five fatal cases reported in 5943 performed PT procedures. This occurred while treating 5517 echinococcal cysts resulting in an overall fatality rate of 0.08% (5 in 5943) and 0.09% (5 in 5517) respectively (Table 2). The overall fatality rate due to lethal anaphylaxis is 0.03% (2 in 5943) and 0.04% (2 in 5517) respectively (Table 2).

Reversible complications were reported in 345 out of 3440 PT procedures (10%) for the treatment of 3232 liver echinococcal cysts (Table 3), in nine out of 175 PT procedures (5%) for the treatment of 142 extra-hepatic echinococcal cysts (Table 5) and in 423 out of 2328 PT procedures (18%) for the treatment of 2143 echinococcal cysts of unspecified anatomical location (Table 4).

In summary, 777 reversible complications were observed in 5943 PT procedures for the treatment of 5517 echinococcal cysts. Therefore, reversible complications were observed in 13% of all PT procedures, corresponding to 14% of all treated echinococcal cysts (Table 6).

The reversible complications fall into three categories: anaphylactic, potentially anaphylactic, and non-anaphylactic:

In total, 99 reversible anaphylactic reactions were reported in 5943 PT procedures for the treatment of 5517 echinococcal cysts. Therefore, reversible allergic reactions complicated 1.7% of all PT procedures, corresponding to 1.8% of all treated echinococcal cysts (Table 7).

The potentially anaphylactic complications include “fever”, “hypotensive reaction”, “vaso-vagal-reaction”, and “nausea and vomiting”. In total, 128 potentially anaphylactic reactions were reported during 5943 PT procedures (2.1%) for the treatment of 5517 (2.3%) echinococcal cysts (Table 6).

Non-anaphylactic complications – ranging from frequently observed “biliary fistulas” to very rare events such as “active arterial hemorrhage”, “intracystic bleeding” or “gallbladder hemorrhage” – were reported in 550 cases during 5943 PT procedures (9.3%) for the treatment of 5517 (10%) echinococcal cysts.

## Discussion

Allergic reactions and anaphylaxis are IgE-mediated immediate hypersensitivity reactions that occur when antigen-specific IgE, bound to Fc receptors on mast cells and basophils, are cross linked by the antigen, activating the cells to rapidly release a variety of mediators such as histamine, enzymes and lipid mediators [28].

While anaphylactic reactions and allergic symptoms are usually observed in cases of treatment-related rupture of echinococcal cysts, they may also occur spontaneously. The symptoms vary from mild urticaria to anaphylactic shock [29]. The presence of specific IgE in serum of patients is a well known feature of CE with levels varying according to cyst number, location, morphology, disease severity, and cyst stage [30], [31].

Despite 75% of CE patients having detectable levels of specific IgE and histamine release by circulating basophils in response to *E. granulosus*, antigens can be detected in 100% of patients [32]. Consequently, allergic reactions are rare and unpredictable. So far, the predictive value of IgE titers (or of IgG4 titers, considered “anti-anaphylactic” isotypes) for the development of allergic reactions has not been investigated.

*Echinococcus* allergens have mainly been studied with the aim of improving the performance of diagnostic tests. Three conserved proteins have been identified (EgEF-1β/δ, EA21 and Eg2HSP70), by screening of an *E. granulosus* cDNA library with IgE from patients with and without cutaneous allergic manifestations showing significantly different IgE-binding reactivity between groups [33], [34], [35]. Nevertheless, the identification of such reactivity by a patient's IgE as a predictive factor for the development of anaphylaxis has never been investigated. Another appealing, still unexplored possibility, is the use of these allergens for desensitization therapy. The control of CE-related allergic reactions relies on the administration of vasoactive agents (e.g. epinephrine) and corticosteroids. Although a study reported less severe hemodynamic alterations in surgical patients pre-treated with histamine H1 plus H2 receptor blockers [36], the usefulness of any pre-operative treatment for the prevention of anaphylaxis has never been demonstrated.

The pathogenesis of anaphylactic reactions in CE is still unclear but commonly explained by the disruption of the integrity of the cyst wall with spillage and translocation of allergenic cyst contents into the host's circulation. Despite this, rupture of echinococcal cysts does not always or necessarily lead to anaphylactic reactions. In a series of 24 patients with proven rupture of echinococcal cysts (12 patients with liver cysts and 12 patients with lung cysts) only four patients (16.7%) had symptoms or a history of allergic reactions [37]. The same observation has been made during surgery of echinococcal cysts, were apparent spillage of cyst fluid – despite all precautions taken – is reported to occur in 5% to 10% of cases, without this necessarily leading to anaphylaxis [38].

In our review, we found an incidence of three anaphylactic fatalities per 10,000 PT procedures (0.03%). To put this figure in perspective, one may consider other conditions where treatment entails the risks of lethal anaphylaxis:

- In the literature, fatal drug reactions are reported to occur in 0.1% of medical inpatients and 0.01% of surgical inpatients (the main drugs implicated are antibiotics and non-steroidal anti-inflammatory drugs) [39].
- Among drug-related allergic reactions, penicillin is one of the drugs about which an abundance of data is available: the rate of serious anaphylactic reactions among patients treated with penicillin ranges from one to four per 10,000 treatment courses [40], [41].
- In a prospective international study to determine the incidence of allergic reactions to monthly intramuscular benzathine penicillin (penicillin G benzathine) injections for the prevention of recurrences of rheumatic fever, 1790 patients from 11 countries were enrolled: 57 of the 1570 patients (3.2%) had an allergic reaction, four (0.2%) had anaphylactic shock and one patient had lethal anaphylaxis (0.05%)[42].
- Allergic reactions to radiographic contrast media are reported to occur in 1% and death in 0.001 - 0.009% of patients [39].

Overall, we found a frequency of 1.67 reversible anaphylactic reactions per 100 PT procedures of echinococcal cysts (1.67%) (Table 7). The majority of these reversible anaphylactic reactions were allergic skin reactions (urticaria, rash, pruritus), reported in 1.1 per 100 PT procedures (1.1%) (Table 7).

Again, to put these figures in perspective, we consulted the literature on drug-related allergic skin reactions: in a large surveillance program on drug-induced allergic cutaneous reactions – including 15,238 consecutive inpatients – Bigby et al. found antibiotics to be associated with the highest risk. Among the 51 drugs studied, allergic cutaneous reactions were observed in 1.8% to 5% of all treated patients (penicillin G: 1.8%, erythromycin: 2%, semisynthetic penicillins: 2.1%, cephalosporins: 2.1%, ampicillin: 3.3%, trimethoprim-sulfamethoxazole: 3.4%, amoxicillin: 5%). In the same study, 2.2% of patients receiving blood products presented allergic cutaneous reactions [43].

One problem with allergic reactions from the puncture or surgery of echinococcal cysts is that the exact pathophysiological cause and correlation with consecutive symptoms remains unclear. Some peri-interventional complications reported as “fever” (111 cases), “hypotensive reaction” (15 cases), “vaso-vagal-reaction” (1 case), and “nausea and vomiting” (1 case) (Table 6) might represent allergic reactions. If this were to be the case, the risk of reversible allergic reactions might be as high as 3.8 per 100 PT procedures (3.8%). Even though some of these cases might represent anaphylactic reactions, it can be assumed that most of the “fever” events (111 of the 128 potentially anaphylactic reactions) are due to infections, as post-interventional “cavity infections” and “abscesses” account for 60 of the total 550 non-allergic reversible complications (Table 6).

Additionally, the concept of anaphylaxis awaits a stricter definition, as there is no consensus on exactly how to define it along with considerable disagreement about its prevalence, diagnosis and management [44], [45].

The retrospective evaluation of publications on PT related complications is certainly limited by a number of factors such as non-uniform definitions of anaphylactic events, the merging of data from different kind of studies – covering different PT methods in different settings and dealing with a different composition of clinical cases – and the denominator issue. Due to the retrospective nature of our review and because we can only analyze published data, a publication bias can also be at work. It can be argued that severe events (e.g. severe anaphylaxis) might be more likely be be published. But one could counter that events assumed to be common (especially the often quoted PT related anaphylaxis) might not as readily be published. Nevertheless, we consider the analysis of the existing published literature a justified approach as no other source of more accurate data is currently available.

Future work in this area is needed to investigate the pathophysiology of anaphylactic reactions in CE and to prospectively study the potential relationship between clinical variables such as location, number, size, stage of the cyst, and risk of anaphylactic reactions. While large, well-designed clinical trials are needed to develop treatment algorithms stratified by cyst stage and available level of health care resources, the analysis of the available literature shows that the traditional fear of lethal anaphylaxis and allergic reactions in PT of echinococcal cysts has been exaggerated by the critics of PT. Provided adequate stand-by resuscitation measures are available, each time an echinococcal cyst is punctured, fear of anaphylactic shock is no longer justified as an argument to avoid this therapeutic option.

A necessary evolution in the clinical management of CE will be the comparative evaluation of different PT and surgical methods in certain situations.

While surgery legitimately maintains a central role in complicated cysts (rupture, biliary fistulas, compression of vital structures, bacterial superinfection, haemorrhage), cysts at high risk of rupture, or large cysts with many daughter vesicles, that are not suitable for percutaneous treatment approaches, PT has shown to be a safe and effective alternative for many patients with suitable cysts. What is needed now are evidence-based criteria to allocate the patient to the most appropriate treatment option according to the specific situation.

## Supporting Information

Table S1 **Percutaneous treatment of liver hydatid cysts.**
(XLS)

Table S2 **Percutaneous treatment of extra-hepatic hydatid cysts.**
(XLS)

References S1 **References of the 124 reviewed publications and references of the excluded publications.**
(DOC)
