# Supplementary material for: Justified Concern or Exaggerated Fear: The Risk of Anaphylaxis in Percutaneous Treatment of Cystic Echinococcosis—A Systematic Literature Review
Source: PLoS Negl Trop Dis. 2011 Jun 14;5(6):e1154. doi: 10.1371/journal.pntd.0001154 (PMC3114754; doi:10.1371/journal.pntd.0001154)
Supplement: References S1 — References of the 124 reviewed publications and references of the excluded publications. (DOC) [file pntd.0001154.s003.doc]

**References of the 124 reviewed publications (in chronological order of publication)**

1. Fornage B (1983) [Fortuitous diagnosis by fine needle puncture under real-time ultrasound control of an atypical hydatid cyst of the liver]. J Radiol 64: 643-645.

2. Claudon M, Chaulieu C, Delgoffe C, Desplechain C, Thomas D, et al. (1984) [Role of echography in the diagnosis and monitoring of hepatic alveolar echinococcosis]. J Radiol 65: 773-780.

3. McCorkell SJ (1984) Unintended percutaneous aspiration of pulmonary echinococcal cysts. AJR Am J Roentgenol 143: 123-126.

4. Livraghi T, Bosoni A, Giordano F, Lai N, Vettori C (1985) Diagnosis of hydatid cyst by percutaneous aspiration: value of electrolyte determinations. J Clin Ultrasound 13: 333-337.

5. Mueller PR, Dawson SL, Ferrucci JT, Jr., Nardi GL (1985) Hepatic echinococcal cyst: successful percutaneous drainage. Radiology 155: 627-628.

6. Ben Amor N, Gargouri M, Gharbi HA, Golvan YJ, Ayachi K, et al. (1986) [Trial therapy of inoperable abdominal hydatid cysts by puncture]. Ann Parasitol Hum Comp 61: 689-692.

7. Bret PM, Fond A, Bretagnolle M, Valette PJ, Thiesse P, et al. (1988) Percutaneous aspiration and drainage of hydatid cysts in the liver. Radiology 168: 617-620.

8. Hira PR, Shweiki H, Lindberg LG, Shaheen Y, Francis I, et al. (1988) Diagnosis of cystic hydatid disease: role of aspiration cytology. Lancet 2: 655-657.

9. Agarwal PK, Husain N, Singh BN (1989) Cytologic findings in aspirated hydatid fluid. Acta Cytol 33: 652-654.

10. Ascoli V, Teggi A, Gossetti F, Nardi F (1990) Hydatid cyst: primary diagnosis by fine-needle aspiration biopsy. Diagn Cytopathol 6: 44-48.

11. Gargouri M, Ben Amor N, Ben Chehida F, Hammou A, Gharbi HA, et al. (1990) Percutaneous treatment of hydatid cysts (Echinococcus granulosus). Cardiovasc Intervent Radiol 13: 169-173.

12. Goffette P, Danneels F, Kurdziel JC, Dondelinger RF (1990) [Diagnosis and percutaneous treatment of pulmonary hydatid cyst. State of the problem apropos of a case]. J Belge Radiol 73: 259-263.

13. Kapila K, Verma K (1990) Aspiration cytology diagnosis of echinococcosis. Diagn Cytopathol 6: 301-303.

14. Pogacnik A, Pohar-Marinsek Z, Us-Krasovec M (1990) Fine needle aspiration biopsy in the diagnosis of liver echinococcosis. Acta Cytol 34: 765-766.

15. al Karawi MA, Mohamed AR, el Tayeb BO, Yasawy MI (1991) Unintentional percutaneous aspiration of a pleural hydatid cyst. Thorax 46: 859-860.

16. Ingram EA, Helikson MA (1991) Echinococcosis (hydatid disease) in Missouri: diagnosis by fine-needle aspiration of a lung cyst. Diagn Cytopathol 7: 527-531.

17. Khuroo MS, Zargar SA, Mahajan R (1991) Echinococcus granulosus cysts in the liver: management with percutaneous drainage. Radiology 180: 141-145.

18. Acunas B, Rozanes I, Celik L, Minareci O, Acunas G, et al. (1992) Purely cystic hydatid disease of the liver: treatment with percutaneous aspiration and injection of hypertonic saline. Radiology 182: 541-543.

19. Giorgio A, Tarantino L, Francica G, Mariniello N, Aloisio T, et al. (1992) Unilocular hydatid liver cysts: treatment with US-guided, double percutaneous aspiration and alcohol injection. Radiology 184: 705-710.

20. Vishnevskii VA, Pomelov VS, Gavrilin AV, Ikramov RZ, Viliavin M (1992) [First experience in treatment of hepatic echinococcal cyst by percutaneous puncture drainage]. Khirurgiia (Mosk): 22-26.

21. Gori S, Campatelli A, Luchi S, Paladini A, Savalli E, et al. (1993) Cytology in the percutaneous treatment of hydatid cysts. A report of four cases. Acta Cytol 37: 423-426.

22. Khuroo MS, Dar MY, Yattoo GN, Zargar SA, Javaid G, et al. (1993) Percutaneous drainage versus albendazole therapy in hepatic hydatidosis: a prospective, randomized study. Gastroenterology 104: 1452-1459.

23. Mittal S, Mangwana S (1993) Primary diagnosis of soft tissue echinococcosis by aspiration cytology. Indian J Pathol Microbiol 36: 298-303.

24. Simonetti G, Profili S, Sergiacomi GL, Meloni GB, Orlacchio A (1993) Percutaneous treatment of hepatic cysts by aspiration and sclerotherapy. Cardiovasc Intervent Radiol 16: 81-84.

25. Akhan O, Ozmen MN, Dincer A, Gocmen A, Kalyoncu F (1994) Percutaneous treatment of pulmonary hydatid cysts. Cardiovasc Intervent Radiol 17: 271-275.

26. Bastid C, Azar C, Doyer M, Sahel J (1994) Percutaneous treatment of hydatid cysts under sonographic guidance. Dig Dis Sci 39: 1576-1580.

27. Sagin HB, Kiroglu Y, Aksoy F (1994) Hydatid cyst of the breast diagnosed by fine needle aspiration biopsy. A case report. Acta Cytol 38: 965-967.

28. Wang X, Li Y, Feng S (1994) [Clinical treatment of hepatic and abdominal hydatid cyst by percutaneous puncture, drainage and curettage]. Zhongguo Ji Sheng Chong Xue Yu Ji Sheng Chong Bing Za Zhi 12: 285-287.

29. Baijal SS, Basarge N, Srinadh ES, Mittal BR, Kumar A (1995) Percutaneous management of renal hydatidosis: a minimally invasive therapeutic option. J Urol 153: 1199-1201.

30. Das DK, Bhambhani S, Pant CS (1995) Ultrasound guided fine-needle aspiration cytology: diagnosis of hydatid disease of the abdomen and thorax. Diagn Cytopathol 12: 173-176.

31. Goel MC, Agarwal MR, Misra A (1995) Percutaneous drainage of renal hydatid cyst: early results and follow-up. Br J Urol 75: 724-728.

32. Saenz-Santamaria J, Moreno-Casado J, Nunez C (1995) Role of fine-needle biopsy in the diagnosis of hydatid cyst. Diagn Cytopathol 13: 229-232.

33. Salama H, Farid Abdel-Wahab M, Strickland GT (1995) Diagnosis and treatment of hepatic hydatid cysts with the aid of echo-guided percutaneous cyst puncture. Clin Infect Dis 21: 1372-1376.

34. Saremi F, McNamara TO (1995) Hydatid cysts of the liver: long-term results of percutaneous treatment using a cutting instrument. AJR Am J Roentgenol 165: 1163-1167.

35. Stoianov G, Grigorov N, Slavov V, Bogusheva E (1995) [Intraoperative anaphylactic shock following the percutaneous puncture of a hepatic echinococcal cyst]. Khirurgiia (Sofiia) 48: 12-13.

36. von Sinner WN, Nyman R, Linjawi T, Ali AM (1995) Fine needle aspiration biopsy of hydatid cysts. Acta Radiol 36: 168-172.

37. Akhan O, Ozmen MN, Dincer A, Sayek I, Gocmen A (1996) Liver hydatid disease: long-term results of percutaneous treatment. Radiology 198: 259-264.

38. Erzurumlu K, Sahin M, Selcuk MB, Yildiz C, Kesim M (1996) Intracystic application of mebendazole solution in the treatment of liver hydatid disease. Preliminary report of two cases. Eur Surg Res 28: 466-470.

39. Kabaalioglu A, Apaydin A, Ozkaynak C, Melikoglu M, Sindel T, et al. (1996) Percutaneous sclerotherapy of a symptomatic simple renal cyst in a child: observation of membrane detachment sign. Eur Radiol 6: 872-874.

40. Dilsiz A, Acikgozoglu S, Gunel E, Dagdonderen L, Koseoglu B, et al. (1997) Ultrasound-guided percutaneous drainage in the treatment of children with hepatic hydatid disease. Pediatr Radiol 27: 230-233.

41. Filice C, Brunetti E (1997) Use of PAIR in human cystic echinococcosis. Acta Trop 64: 95-107.

42. Khuroo MS, Wani NA, Javid G, Khan BA, Yattoo GN, et al. (1997) Percutaneous drainage compared with surgery for hepatic hydatid cysts. N Engl J Med 337: 881-887.

43. Mawhorter S, Temeck B, Chang R, Pass H, Nash T (1997) Nonsurgical therapy for pulmonary hydatid cyst disease. Chest 112: 1432-1436.

44. Stefaniak J (1997) Fine needle aspiration biopsy in the differential diagnosis of the liver cystic echinococcosis. Acta Trop 67: 107-111.

45. Akhan O, Bilgic S, Akata D, Kiratli H, Ozmen MN (1998) Percutaneous treatment of an orbital hydatid cyst: a new therapeutic approach. Am J Ophthalmol 125: 877-879.

46. Akhan O, Üstünsöz B, Somuncu İ, Özmen M, Öner A, et al. (1998) Percutaneous renal hydatid cyst treatment: long-term results. Abdominal Imaging 23: 209-213.

47. Anadol D, Gocmen A, Kiper N, Ozcelik U (1998) Hydatid disease in childhood: a retrospective analysis of 376 cases. Pediatr Pulmonol 26: 190-196.

48. Mikic D, Trnjak Z, Bojic I, Begovic V, Stankovic N, et al. (1998) [Personal experience in the diagnosis and treatment of hepatic echinococcosis]. Vojnosanit Pregl 55: 489-499.

49. Salama HM, Ahmed NH, el Deeb N, Ahmed R (1998) Hepatic hydatid cysts: sonographic follow-up after percutaneous sonographically guided aspiration. J Clin Ultrasound 26: 455-460.

50. Tan A, Yakut M, Kaymakcioglu N, Ozerhan IH, Cetiner S, et al. (1998) The results of surgical treatment and percutaneous drainage of hepatic hydatid disease. Int Surg 83: 314-316.

51. Campatelli A, Luchi S, Di Candio G, Messina F, Di Vito A, et al. (1999) PAIR or PAI as percutaneous therapy of hepatic cystic hydatid disease: report of ten cases. Puncture, aspiration, injection, re-aspiration. Ital J Gastroenterol Hepatol 31: 426.

52. Fava C, Patetta R, Cozzi L, Assi A (1999) [Renal hydatid cyst: cytological diagnosis using fine needle biopsy (FNA)]. Pathologica 91: 115-118.

53. Firpi RJ, Lozada LR, Torres EA, Villamarzo G, Lobera A (1999) Fine-needle aspiration diagnosis of hydatid cyst. P R Health Sci J 18: 129-131.

54. Men S, Hekimoglu B, Yucesoy C, Arda IS, Baran I (1999) Percutaneous treatment of hepatic hydatid cysts: an alternative to surgery. AJR Am J Roentgenol 172: 83-89.

55. Nepka C, Kafanas A, Manatakis K (1999) Fine needle aspiration cytology in the diagnosis of muscular echinococcosis. Acta Cytol 43: 512-513.

56. Pelaez V, Kugler C, del Carpio M, Correa D, Lopez E, et al. (1999) [Treatment of hepatic hydatid cysts by percutaneous aspiration and hypertonic saline injection: results of a cooperative work]. Bol Chil Parasitol 54: 63-69.

57. Singh A, Singh Y, Sharma VK, Agarwal AK, Bist D (1999) Diagnosis of hydatid disease of abdomen and thorax by ultrasound guided fine needle aspiration cytology. Indian J Pathol Microbiol 42: 155-156.

58. Ustunsoz B, Akhan O, Kamiloglu MA, Somuncu I, Ugurel MS, et al. (1999) Percutaneous treatment of hydatid cysts of the liver: long-term results. AJR Am J Roentgenol 172: 91-96.

59. Ustunsoz B, Alemdaroglu A, Bulakbasi N, Uzar AI, Duru NK (1999) Percutaneous treatment of hepatic hydatid cyst in pregnancy. Arch Gynecol Obstet 262: 181-184.

60. Valverde C, Lam J, Ibanez P, Cruzat C (1999) [Neck hydatidosis. Thyroid and submaxillary gland involvement in 2 cases]. Rev Med Chil 127: 1108-1111.

61. Yattoo GN, Khuroo MS, Zargar SA, Bhat FA, Sofi BA (1999) Case report: Percutaneous drainage of the pancreatic head hydatid cyst with obstructive jaundice. J Gastroenterol Hepatol 14: 931-934.

62. Bosanac ZB, Lisanin L (2000) Percutaneous drainage of hydatid cyst in the liver as a primary treatment: review of 52 consecutive cases with long-term follow-up. Clin Radiol 55: 839-848.

63. Grigorov N, Golemanov B, Mitova R, Genov I, Damianov D, et al. (2000) [Percutaneous puncture treatment of hepatic cystic echinococcosis under ultrasound guidance]. Khirurgiia (Sofiia) 56: 28-31.

64. Haddad MC, Sammak BM, Al-Karawi M (2000) Percutaneous treatment of heterogenous predominantly solid echopattern echinococcal cysts of the liver. Cardiovasc Intervent Radiol 23: 121-125.

65. Kabaalioglu A, Karaali K, Apaydin A, Melikoglu M, Sindel T, et al. (2000) Ultrasound-guided percutaneous sclerotherapy of hydatid liver cysts in children. Pediatr Surg Int 16: 346-350.

66. Odev K, Paksoy Y, Arslan A, Aygun E, Sahin M, et al. (2000) Sonographically guided percutaneous treatment of hepatic hydatid cysts: long-term results. J Clin Ultrasound 28: 469-478.

67. Pelaez V, Kugler C, Correa D, Del Carpio M, Guangiroli M, et al. (2000) PAIR as percutaneous treatment of hydatid liver cysts. Acta Trop 75: 197-202.

68. Aygun E, Sahin M, Odev K, Vatansev C, Aksoy F, et al. (2001) The management of liver hydatid cysts by percutaneous drainage. Can J Surg 44: 203-209.

69. Brunetti E, Filice C (2001) Radiofrequency thermal ablation of echinococcal liver cysts. Lancet 358: 1464.

70. Gurkan OU, Topu Z, Ozdemir N, Ekinci C, Numanoglu N (2001) Repeated unintentional transthoracic needle aspiration of a pulmonary hydatid cyst. Scand J Infect Dis 33: 941-942.

71. Haddad MC, Al-Awar G, Huwaijah SH, Al-Kutoubi AO (2001) Echinococcal cysts of the liver: a retrospective analysis of clinico-radiological findings and different therapeutic modalities. Clin Imaging 25: 403-408.

72. Handa U, Mohan H, Ahal S, Mukherjee KK, Dabra A, et al. (2001) Cytodiagnosis of hydatid disease presenting with Horner's syndrome: a case report. Acta Cytol 45: 784-788.

73. Mikic D, Bojic I, Sjenicic G, Djurcic P, Djokic M, et al. (2001) [Successful treatment of echinococcal kidney disease with a combination of albendazole administration and percutaneous drainage]. Vojnosanit Pregl 58: 317-321.

74. Ormeci N, Soykan I, Bektas A, Sanoglu M, Palabiyikoglu M, et al. (2001) A new percutaneous approach for the treatment of hydatid cysts of the liver. Am J Gastroenterol 96: 2225-2230.

75. Sayek I, Onat D (2001) Diagnosis and treatment of uncomplicated hydatid cyst of the liver. World J Surg 25: 21-27.

76. Sinha R, Sharma N (2001) Abdominal hydatids: a minimally invasive approach. JSLS 5: 237-240.

77. Akhan O, Ensari S, Ozmen M (2002) Percutaneous treatment of a parotid gland hydatid cyst: a possible alternative to surgery. Eur Radiol 12: 597-599.

78. Das DK, Choudhury U (2002) Hydatid disease: an unusual breast lump. J Indian Med Assoc 100: 327-328.

79. Dede S, Dede H, Caliskan E, Demir B (2002) Recurrent pelvic hydatid cyst obstructing labor, with a concomitant hepatic primary. A case report. J Reprod Med 47: 164-166.

80. Duta C, Pascut M, Bordos D (2002) [Percutaneous treatment of the liver hydatid cysts under sonographic guidance]. Chirurgia (Bucur) 97: 173-177.

81. Dwivedi M, Misra SP, Dwivedi S, Kumar S, Misra V (2002) Percutaneous treatment of hepatic hydatid cysts using hypertonic saline. J Assoc Physicians India 50: 647-650.

82. Gavrilin AV, Kuntsevich GI, Vishnevskii VA, Ikramov RZ, Zhurenkova TV, et al. (2002) [Ultrasound-assisted puncture method of treatment of hepatic hydatid cysts]. Khirurgiia (Mosk): 39-46.

83. Giordano A, Santagata A, Di Meo E, Della Corte L, Di Robbio R (2002) [Alcohol treatment of hydatid cyst in a case of old hydatidosis with hepatic skip]. Minerva Chir 57: 503-505.

84. Melis M, Marongiu L, Scintu F, Pisano M, Capra F, et al. (2002) Primary hydatid cysts of psoas muscle. ANZ J Surg 72: 443-445.

85. Natarajan A, Rozario A (2002) Percutaneous expulsion of hydatid liver cyst following sclerotherapy. Indian J Gastroenterol 21: 36-37.

86. Ormeci N, Soykan I, Palabiyikoglu M, Idilman R, Erdem H, et al. (2002) A new therapeutic approach for treatment of hydatid cysts of the spleen. Dig Dis Sci 47: 2037-2044.

87. Polat K, Balik A, Oren D (2002) Percutaneous drainage of hydatid cyst of the liver: long-term results. HPB (Oxford) 4: 163-166.

88. Rajagopal KV, Bishwas R (2002) Hydatid cyst of the liver presenting as an inferior vena cava obstruction. J Clin Ultrasound 30: 114-116.

89. Schipper HG, Lameris JS, van Delden OM, Rauws EA, Kager PA (2002) Percutaneous evacuation (PEVAC) of multivesicular echinococcal cysts with or without cystobiliary fistulas which contain non-drainable material: first results of a modified PAIR method. Gut 50: 718-723.

90. Sinan T, Sheikh M, Chisti FA, Al Saeed O, Sheikh Z, et al. (2002) Diagnosis of abdominal hydatid cyst disease: the role of ultrasound and ultrasound-guided fine needle aspiration cytology. Med Princ Pract 11: 190-195.

91. Sodhani P, Gupta S, Jain S (2002) Unusual presentation of disseminated echinococcosis with thyroid involvement: diagnosis by fine needle aspiration cytology in a clinically unsuspected case. Acta Cytol 46: 75-76.

92. Vuitton DA ZWX, Li Feng S, Sheng Chen J, Shou Li Y, Li SF, Ke Tang Q (2002) PAIR-derived US-guided techniques for the treatment of cystic echinococcosis: a Chinese experience. GUT e-letter.

93. Brigic E, Zerem E, Terzic S (2003) [Ultrasonographic guidance of percutaneous drainage as a new method of treatment of echinococcal cysts]. Med Arh 57: 13-15.

94. Geramizadeh B, Boob R, Talei AR, Rasekhi A (2003) Fine needle aspiration cytology in hydatid cyst of the breast. Acta Cytol 47: 701-702.

95. Gokce C, Patiroglu T, Aksehirli S, Durak AC, Kelestimur F (2003) Hydatic cyst in the thyroid gland diagnosed by fine-needle aspiration biopsy. Thyroid 13: 987-989.

96. Khazim R, Fares Y, Heras-Palou C, Ruiz Barnes P (2003) Posterior decompression of spinal hydatidosis: long term results: Fundacion Jimenez Diaz, Madrid, Spain. Clin Neurol Neurosurg 105: 209-214.

97. Rauhofer U, Prager G, Hormann M, Auer H, Kaserer K, et al. (2003) Cystic echinococcosis of the thyroid gland in children and adults. Thyroid 13: 497-502.

98. Saenz-Santamaria J, Catalina-Fernandez I, Fernandez de Mera JJ (2003) Hydatid cyst in soft tissues mimicking malignant tumors. Diagnosis by fine needle aspiration cytology. Acta Cytol 47: 337-340.

99. Zerem E, Sabanovic Z, Smajic M (2003) [Percutaneous treatment of abdominal and retroperitoneal echinococcal cysts using ultrasonography]. Med Arh 57: 71-73.

100. Etlik O, Arslan H, Bay A, Sakarya ME, Harman M, et al. (2004) Abdominal hydatid disease: long-term results of percutaneous treatment. Acta Radiol 45: 383-389.

101. Bastid C, Ayela P, Sahel J (2005) Percutaneous treatment of a complex hydatid cyst of the liver under sonographic control. Report of the first case. Gastroenterol Clin Biol 29: 191-192.

102. Gabal AM, Khawaja FI, Mohammad GA (2005) Modified PAIR technique for percutaneous treatment of high-risk hydatid cysts. Cardiovasc Intervent Radiol 28: 200-208.

103. Goktay AY, Secil M, Gulcu A, Hosgor M, Karaca I, et al. (2005) Percutaneous treatment of hydatid liver cysts in children as a primary treatment: long-term results. J Vasc Interv Radiol 16: 831-839.

104. Ormeci N, Idilman R, Tuzun A, Erdem H, Palabiyikoglu M (2005) A new percutaneous approach for the treatment of hydatid cyst of the kidney: long-term follow-up. Int Urol Nephrol 37: 461-464.

105. Paksoy Y, Odev K, Sahin M, Arslan A, Koc O (2005) Percutaneous treatment of liver hydatid cysts: comparison of direct injection of albendazole and hypertonic saline solution. AJR Am J Roentgenol 185: 727-734.

106. Yagci G, Ustunsoz B, Kaymakcioglu N, Bozlar U, Gorgulu S, et al. (2005) Results of surgical, laparoscopic, and percutaneous treatment for hydatid disease of the liver: 10 years experience with 355 patients. World J Surg 29: 1670-1679.

107. Zerem E, Nuhanovic A, Caluk J (2005) Modified pair technique for treatment of hydatid cysts in the spleen. Bosn J Basic Med Sci 5: 74-78.

108. Battyany I, Herbert Z, Rostas T, Vincze A, Fulop A, et al. (2006) Successful percutaneous drainage of a giant hydatid cyst in the liver. World J Gastroenterol 12: 812-814.

109. Iyilikci L, Balkan BK, Capar E (2006) Sedation for percutaneous treatment of hepatic hydatid cyst in a pregnant patient. Arch Gynecol Obstet 274: 113-114.

110. Kabaalioglu A, Ceken K, Alimoglu E, Apaydin A (2006) Percutaneous imaging-guided treatment of hydatid liver cysts: do long-term results make it a first choice? Eur J Radiol 59: 65-73.

111. Men S, Yucesoy C, Edguer TR, Hekimoglu B (2006) Percutaneous treatment of giant abdominal hydatid cysts: long-term results. Surg Endosc 20: 1600-1606.

112. Yilmaz Y, Kosem M, Ceylan K, Koseoglu B, Yalcinkaya I, et al. (2006) Our experience in eight cases with urinary hydatid disease: a series of 372 cases held in nine different clinics. Int J Urol 13: 1162-1165.

113. Zerem E, Jusufovic R (2006) Percutaneous treatment of univesicular versus multivesicular hepatic hydatid cysts. Surg Endosc 20: 1543-1547.

114. Ormeci N, Idilman R, Akyar S, Palabiyikoglu M, Coban S, et al. (2007) Hydatid cysts in muscle: a modified percutaneous treatment approach. Int J Infect Dis 11: 204-208.

115. Sakaguchi H, Tanaka T, Marugami N, Kichikawa K, Horiuchi H, et al. (2007) Cystic echinococcosis in immigrant from Peru: first case treated with percutaneous treatment in Japan. Parasitol Int 56: 207-210.

116. Fisichella PM, Donaldson K, Helton WS (2008) Hepatic and splenic hydatidosis managed with percutaneous aspiration, injection, and reaspiration (PAIR) of the hepatic cyst and laparoscopic splenectomy. J Gastrointest Surg 12: 1615-1617.

117. Loutfi SI, Arabi MS, Safadi BY, Haddad MC (2008) Life-threatening liver laceration with arterial hemorrhage complicating percutaneous treatment of hepatic hydatid cyst. J Med Liban 56: 185-188.

118. Ustunsoz B, Ugurel MS, Uzar AI, Duru NK (2008) Percutaneous treatment of hepatic hydatid cyst in pregnancy: long-term results. Arch Gynecol Obstet 277: 547-550.

119. Akhan O, Canyigit M, Kaya D, Koksal A, Akgoz A, et al. (2009) Long-Term Follow-Up of the Percutaneous Treatment of Hydatid Cyst in the Adrenal Gland: A Case Report and Review of the Literature. Cardiovasc Intervent Radiol.

120. Aribas BK, Dingil G, Koroglu M, Ungul U, Zarali AC (2009) Liver Hydatid Cyst with Transdiaphragmatic Rupture and Lung Hydatid Cyst Ruptured into Bronchi and Pleural Space. Cardiovasc Intervent Radiol.

121. Avcu S, Unal O, Kotan C, Ozturk M, Ozen O (2009) Submandibular and thyroid gland involvement of hydatid cysts: a very rare association with percutaneous treatment. Diagn Interv Radiol.

122. Bilgic S, Kose O, Sehirlioglu A, Safaz I, Ozkan H (2009) Primary paraspinal hydatid cyst treated with puncture, aspiration, injection and re-aspiration (PAIR) technique: a case report. Eur Spine J 18 Suppl 2: 165-167.

123. Giorgio A, Di Sarno A, de Stefano G, Liorre G, Farella N, et al. (2009) Sonography and clinical outcome of viable hydatid liver cysts treated with double percutaneous aspiration and ethanol injection as first-line therapy: efficacy and long-term follow-up. AJR Am J Roentgenol 193: W186-192.

124. Park KH, Jung SI, Jang HC, Shin JH (2009) First successful puncture, aspiration, injection, and re-aspiration of hydatid cyst in the liver presenting with anaphylactic shock in Korea. Yonsei Med J 50: 717-720.

**References of the excluded publications (covering identical cases)**

1. Giorgio A. et al. (1991) Percutaneous therapy of hydatid cyst of the liver with ultrasound-guided double puncture-aspiration and alcoholization. Radiol Med 82(4):460-4

*Reason for exclusion (1)*: the included publication [Giorgio A. et al. (1992) Unilocular hydatid liver cysts: treatment with US-guided, double percutaneous aspiration and alcohol injection. Radiology 184: 705-710.] covers identical cases.

1. Giorgio A. et al. (2001) Hydatid liver cyst: an 11-year experience of treatment with percutaneous aspiration and ethanol injection. J Ultrasound Med 20(12):1377-9
2. Giorgio A. et al. (2003) Complications after interventional sonography of focal liver lesions: a 22-year single-center experience. J Ultrasound Service 22(2):193-205
3. Giorgio A. et al. (2008) Long term results of percutaneous treatment of hydatid liver cysts: a single center 17 years experience. Infection 36; 256-261

*Reason for exclusion (2,3,4):* the included publication [Giorgio A. et al. (2009) Sonography and clinical outcome of viable hydatid liver cysts treated with double percutaneous aspiration and ethanol injection as first-line therapy: efficacy and long-term follow-up. AJR Am J Roentgenol 193: W186-192.] covers identical cases.
